# Supplementary material for: Impaired excitability of fast-spiking neurons in a novel mouse model of KCNC1 epileptic encephalopathy
Source: eLife. 2026 Feb 18;13:RP103784. doi: 10.7554/eLife.103784 (PMC12916103; doi:10.7554/eLife.103784)
Supplement: MDAR checklist [file elife-103784-mdarchecklist1.pdf]

Westbrook Centre
Milton Road
Cambridge CB4 1YG, UK
P 01223 855340
W elifesciences.org
T @elife

Materials Design Analysis Reporting (MDAR)

Checklist for Authors

The MDAR framework establishes a minimum set of requirements in transparent reporting mainly applicable to studies in the life sciences.

eLife asks authors to **provide detailed information within their article** to facilitate the interpretation and replication of their work. Authors can also upload supporting materials to comply with relevant reporting guidelines for health-related research (see EQUATOR Network), life science research (see the BioSharing Information Resource), or animal research (see the ARRIVE Guidelines and the STRANGE Framework; for details, see *eLife*'s Journal Policies). Where applicable, authors should refer to any relevant reporting standards materials in this form.

For all that apply, please note **where in the article** the information is provided. Please note that we also collect information about data availability and ethics in the submission form.

Materials:

**Newly created materials**	**Indicate where provided: section/figure legend**	**N/A**
The manuscript includes a dedicated "materials availability statement" providing transparent disclosure about availability of newly created materials including details on how materials can be accessed and describing any restrictions on access.	Methods section	

**Antibodies**	**Indicate where provided: section/figure legend**	**N/A**
rabbit anti-Kv3.1b, Alomone Labs APC-014, RRID:AB_2040166<br>mouse anti-parvalbumin, EMD Millipore MAB1572, RRID:AB_2174013	Methods > Immunohistochemistry section	

**DNA and RNA sequences**	**Indicate where provided: section/figure legend**	**N/A**
		N/A

**Cell materials**	**Indicate where provided: section/figure legend**	**N/A**
Cell lines:<br>HEK-293T (ATCC, CRL-3216; RRID:CVCL_0063).	Methods > Plasmid preparation, cell culture and transfection section	
		N/A

Experimental animals	Indicate where provided: section/figure legend	N/A
Laboratory animals or Model organisms: Mouse<br>Pvalb-tdTomato RRID:IMSR_JAX:027395<br>ACTB-Cre RRID:IMSR_JAX:003376	Methods > Experimental animals section	N/A
Animal observed in or captured from the fie	Methods > Plasmid preparation, cell culture and transfection section	N/A

Plants and microbes	Indicate where provided: section/figure legend	N/A
Plants		N/A
Microbes		N/A

Human research participants	Indicate where provided: section/figure legend) or state if these demographics were not collected	N/A
		N/A

Design:

Study protocol	Indicate where provided: section/figure legend	N/A
If the study protocol has been pre-registered, provide DOI. For clinical trials, provide the trial registration number OR cite DOI.		

Laboratory protocol	Indicate where provided: section/figure legend	N/A
Provide DOI OR other citation details if detailed step-by-step protocols are available.		

**Experimental study design (statistics details) ***		
**For in vivo studies: State whether and how the following have been done**	**Indicate where provided: section/figure legend. If it could have been done, but was not, write "not done"**	**N/A**
Sample size determination	Sample size was determined using a-priori power analysis based on a previous study that used the same experimental task, please see section *Materials and Methods*, sub-section *Participants*.	
Randomisation	The order of drug sessions (including placebo) was counterbalanced across subjects to ensure comparable numbers of the same sequence of sessions (six different sequences) within the sample of 30 participants, please see section *Materials and Methods*, sub-section *Pharmacological manipulations*.	
Blinding	Pharmacological manipulations followed a double-blind cross-over design. Unblinding was only done once all data had been acquired. Please see section *Materials and Methods*, sub-section *Pharmacological manipulations*.	
Inclusion/exclusion criteria	Exclusion criteria were conditions or behaviors that could affect either pain perception or reinforcement learning and are listed in the section *Materials and Methods*, sub-section *Participants*.	

**Sample definition and in-laboratory replication**	**Indicate where provided: section/figure legend**	**N/A**
State number of times the experiment was replicated in the laboratory.		N/A
Define whether data describe technical or biological replicates.		N/A

**Ethics**	**Indicate where provided: section/submission form**	**N/A**
Studies involving human participants: State details of authority granting ethics approval (IRB or equivalent committee(s), provide reference number for approval.	The study was approved by the Ethics Committee of the Medical Faculty Mannheim, Heidelberg University, (approval reference 2014-504N-MA)	

	and written informed consent was obtained from all participants prior to participation according to the revised Declaration of Helsinki (World Medical Association, 2013).	
Studies involving experimental animals: State details of authority granting ethics approval (IRB or equivalent committee(s), provide reference number for approval.		N/A
Studies involving specimen and field samples: State if relevant permits obtained, provide details of authority approving study; if none were required, explain why.		N/A

**Dual Use Research of Concern (DURC)**	**Indicate where provided: section/submission form**	**N/A**
If study is subject to dual use research of concern regulations, state the authority granting approval and reference number for the regulatory approval.		N/A

Analysis:

**Attrition**	**Indicate where provided: section/figure legend**	**N/A**
Describe whether exclusion criteria were pre-established. Report if sample or data points were omitted from analysis. If yes, report if this was due to attrition or intentional exclusion and provide justification.	Exclusion criteria for the study participation were pre-established. Data from two out of thirty participants were excluded from the statistical analysis, one due to failure to comply with the experimental procedures and one due to technical failure of the equipment. For one participant data of one session (levodopa) are missing due to drop-out. For bivariate correlations we excluded multivariate outliers (n=1) by comparing squared Mahalanobis distances to a χ^2 distribution. Please see section *Materials and Methods*, sub-section *Statistical analysis*	N/A

**Statistics**	**Indicate where provided: section/figure legend**	**N/A**
Describe statistical tests used and justify choice of tests.	Statistical tests are described in detail in the section *Methods and materials*, sub-section *Statistical analysis*. We used linear and logistic mixed-effects models to analyze behavioral data because these models can account for both, the repeated measures structure of the data and the imbalanced structure due	N/A

	to the probabilistic reward schedule. We used Hierarchical Bayesian modelling to fit reward learning models to the data. Hierarchical models estimate group and individual parameters simultaneously to mutually inform and constrain each other, which yields reliable estimates for both, individual and group level parameters.	

**Data availability**	**Indicate where provided: section/submission form**	**N/A**
For newly created and reused datasets, the manuscript includes a data availability statement that provides details for access (or notes restrictions on access).	Behavioral and questionnaire data is available as csv file at the project's Open Science Framework page (osf.io/5xjt9).	
When newly created datasets are publicly available, provide accession number in repository OR DOI and licensing details where available.	See above	
If reused data is publicly available provide accession number in repository OR DOI, OR URL, OR citation.		N/A

**Code availability**	**Indicate where provided: section/figure legend**	**N/A**
For any computer code/software/mathematical algorithms essential for replicating the main findings of the study, whether newly generated or re-used, the manuscript includes a data availability statement that provides details for access or notes restrictions.	All software used for the analyses is open source and publicly available. All software packages that were used are named and cited in the section *Materials and Methods*, sub-sections *Statistical analysis* and *Estimation of prediction errors and their role in endogenous pain modulation*.	
Where newly generated code is publicly available, provide accession number in repository, OR DOI OR URL and licensing details where available. State any restrictions on code availability or accessibility.		N/A
If reused code is publicly available provide accession number in repository OR DOI OR URL, OR citation.		N/A

Reporting:

The MDAR framework recommends adoption of discipline-specific guidelines, established and endorsed through community initiatives.

**Adherence to community standards**	**Indicate where provided: section/figure legend**	**N/A**

State if relevant guidelines (e.g., ICMJE, MIBBI, ARRIVE, STRANGE) have been followed, and whether a checklist (e.g., CONSORT, PRISMA, ARRIVE) is provided with the manuscript.		N/A

---

* We provide the following guidance regarding transparent reporting and statistics; we also refer authors to Ten common statistical mistakes to watch out for when writing or reviewing a manuscript.

Sample-size estimation

- You should state whether an appropriate sample size was computed when the study was being designed
- You should state the statistical method of sample size computation and any required assumptions
- If no explicit power analysis was used, you should describe how you decided what sample (replicate) size (number) to use

Replicates

- You should report how often each experiment was performed
- You should include a definition of biological versus technical replication
- The data obtained should be provided and sufficient information should be provided to indicate the number of independent biological and/or technical replicates
- If you encountered any outliers, you should describe how these were handled
- Criteria for exclusion/inclusion of data should be clearly stated
- High-throughput sequence data should be uploaded before submission, with a private link for reviewers provided (these are available from both GEO and ArrayExpress)

Statistical reporting

- Statistical analysis methods should be described and justified
- Raw data should be presented in figures whenever informative to do so (typically when N per group is less than 10)
- For each experiment, you should identify the statistical tests used, exact values of N, definitions of center, methods of multiple test correction, and dispersion and precision measures (e.g., mean, median, SD, SEM, confidence intervals; and, for the major substantive results, a measure of effect size (e.g., Pearson's r, Cohen's d)
- Report exact p-values wherever possible alongside the summary statistics and 95% confidence intervals. These should be reported for all key questions and not only when the p-value is less than 0.05.

Group allocation

- Indicate how samples were allocated into experimental groups (in the case of clinical studies, please specify allocation to treatment method); if randomization was used, please also state if restricted randomization was applied
- Indicate if masking was used during group allocation, data collection and/or data analysis
